# Development and validation of a simulation-based assessment tool in colonoscopy

**DOI:** 10.1186/s41077-023-00260-5

**Published:** 2023-08-10

**Authors:** Claudia Jaensch, Rune D. Jensen, Charlotte Paltved, Anders H. Madsen

**Affiliations:** 1 Surgical Research Department, Regional Hospital Gødstrup, Herning, Denmark; 2 Department of Clinical Medicine, Aarhus University, Aarhus, Denmark; 3 Corporate HR MidtSim, Central Region of Denmark, Aarhus, Denmark; 4 Surgical Department, Regional Hospital Gødstrup, Herning, Denmark

**Keywords:** Simulation, Simulation-based training, Colonoscopy, Competency assessment, Patient safety

## Abstract

**Background:**

Colonoscopy is difficult to learn. Virtual reality simulation training is helpful, but how and when novices should progress to patient-based training has yet to be established. To date, there is no assessment tool for credentialing novice endoscopists prior to clinical practice. The aim of this study was to develop such an assessment tool based on metrics provided by the simulator. The metrics used for the assessment tool should be able to discriminate between novices, intermediates, and experts and include essential checklist items for patient safety.

**Methods:**

The validation process was conducted based on the Standards for Educational and Psychological Testing. An expert panel decided upon three essential checklist items for patient safety based on Lawshe’s method: perforation, hazardous tension to the bowel wall, and cecal intubation. A power calculation was performed. In this study, the Simbionix GI Mentor II simulator was used. Metrics with discriminatory ability were identified with variance analysis and combined to form an aggregate score. Based on this score and the essential items, pass/fail standards were set and reliability was tested.

**Results:**

Twenty-four participants (eight novices, eight intermediates, and eight expert endoscopists) performed two simulated colonoscopies. Four metrics with discriminatory ability were identified. The aggregate score ranged from 4.2 to 51.2 points. Novices had a mean score of 10.00 (SD 5.13), intermediates 24.63 (SD 7.91), and experts 30.72 (SD 11.98). The difference in score between novices and the other two groups was statistically significant (*p*<0.01). Although expert endoscopists had a higher score, the difference was not statistically significant (*p*=0.40). Reliability was good (Cronbach’s alpha=0.86). A pass/fail score was defined at 17.1 points with correct completion of three essential checklist items, resulting in three experts and three intermediates failing and one novice passing the assessment.

**Conclusion:**

We established a valid and reliable assessment tool with a pass/fail standard on the simulator. We suggest using the assessment after simulation-based training before commencing work-based learning.

## Validating a simulation-based assessment tool in colonoscopy 

### Background

Colonoscopy is the gold standard in diagnosis and treatment of colorectal cancer. It is a complex procedure and difficult to learn. Several hundred procedures must be performed to gain proficiency [1, 2]. Ward et al. found that 233 procedures are required to achieve an acceptable quality standard [3]. 

Training in colonoscopy is traditionally performed on patients under expert supervision. This approach may cause distress to the patient and is time-consuming. Simulation-based training provides a risk-free environment for the trainee without compromising patient safety. Simulation-based training in colonoscopy is recognized as an effective supplement to bedside training [3] and can be of use to speed up early colonoscopy training on patients [4]. 

Questions remain with regard to how and when novices can progress from early simulation-based colonoscopy to workplace-based training on patients. To date, there is no simulation-based assessment tool available for credentialing novice endoscopists, including both performance metrics and patient safety items. 

Several studies have demonstrated the Simbionix colonoscopy simulator’s ability to discriminate between novices and more experienced endoscopists [5–8]. However, in all of these studies, critical issues need to be addressed: In one study, both gastroscopy and colonoscopy cases were investigated [6]. Another study only included two metrics in their assessment [7], and a third study included three groups, which only differed very slightly in experience [8]. Only one of the abovementioned studies created credible pass/fail standards for assessment in a simulation-based training program [5]; yet, critical action items like occurrence of perforation or hazardous tension to the bowel wall were not taken into account. These items are of profound importance for patient safety and should be taken into account when standard setting is performed [9].

This study aims at developing an assessment tool based on metrics provided by the simulator and several patient safety items.

To determine when the trainee is competent for workplace-based learning, the following questions must be answered: (1) Are the metrics in the colonoscopy simulator able to discriminate between novices, intermediates, and experienced endoscopists? (2) If so, can that ability be used for competency-based assessment? (3) What score should be used as a benchmark to ensure mastery of the procedure and patient safety?

In order to answer these questions, we investigated proficiency levels according to physician competencies. The aim of this study was to investigate the Simbionix colonoscopy simulator’s ability to discriminate between three different levels of competency (novice, intermediate, and expert) and to develop a simulation-based assessment tool depending on procedural data and essential checklist items. Hence, the assessment tool contains two steps: (1) an aggregate score based on two cases and (2) correct completion of essential checklist items.

## Material and methods

### Participants

**Fig. 1:**
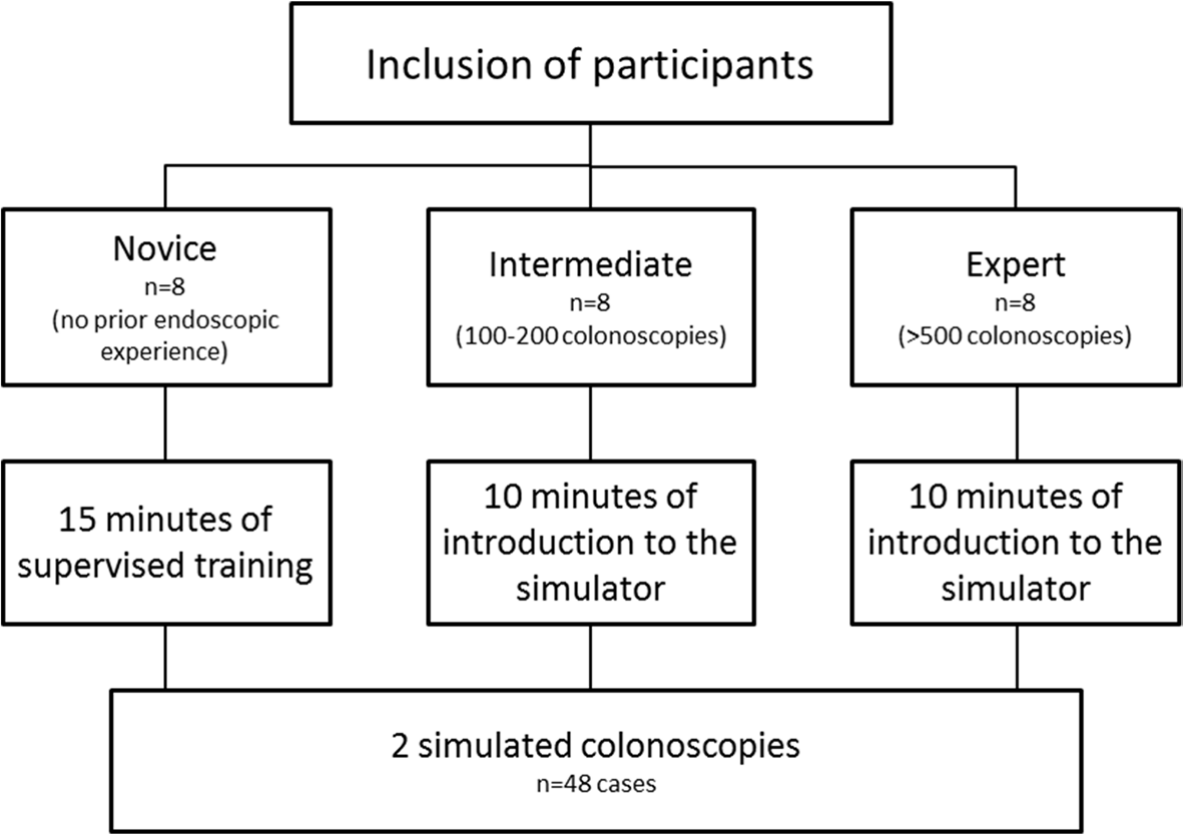
The flowchart shows the study design. Inclusion of the 24 participants into three study groups, introduction to the simulator, and performance of simulated colonoscopies

Three groups of participants were included in this study. Group one consisted of novices recruited among medical students without prior endoscopy experience. Group two consisted of endoscopists with intermediate experience who had performed between 100 and 200 colonoscopies. In group three, expert endoscopists with more than 5 years’ experience and more than 500 performed colonoscopies participated. Participants in groups two and three were recruited from fellows in gastroenterology, surgery or expert nurse endoscopists (see study flowchart in Fig. 1). Eligible candidates for the three groups were approached and asked for participation in the study.

All participants filled out a questionnaire with demographic data such as gender, age, specialty (medical student, fellow in gastroenterology or surgery, and nurse endoscopist), number of colonoscopies performed overall and during the past 12 months, prior experience with simulation-based colonoscopy and, finally, participation in colonoscopy courses.

### Simulated colonoscopies

In this study, the GI Mentor II simulator (Simbionix Corporation, Cleveland, OH) was used. This simulator provides a series of cases with increasing complexity including pathology. To ensure consistency in measurements and increase reliability of the results, all participants were requested to perform two simulated cases. Both cases represented a standard colonoscopy; however, the second case was slightly more difficult in terms of loop formation.

Since evaluating more complex scenarios increases the chance of detecting a difference in competency [10], two cases (case four and seven from the lower gastrointestinal (GI) module one) were chosen based on difficulty and clinical relevance. This was done by an expert panel consisting of two experienced surgical endoscopists (CJ, AHM), one associate professor in medical education (RDJ) and one associate professor in simulation who was also the medical director of a simulation centre (CP).

### Essential checklist items

After each procedure, the simulator generated the following eight metrics: percentage of mucosa visualized, time to reach the cecum, intubation of the terminal ileum, time with clear view, time spent on looping, time spent with “patient” in discomfort, hazardous tension to the bowel wall, and occurrence of perforation.

The relevance of these metrics was discussed in the abovementioned expert panel. It was decided to exclude the metric patient discomfort due to the following flaw in discomfort measurement in simulated colonoscopy: The simulator measures discomfort by means of two independent factors: loop formation and insufflation. During loop formation, the extension of the bowel and mesentery, which results in discomfort, is simulated based on advancement of the endoscope as it would happen in a real-life colonoscopy. Regarding insufflation, however, a mechanism different from that of real-life colonoscopy is used. In a real colonoscopy, the middle finger of the endoscopist’s left hand needs to obstruct the air channel on the insufflation button completely, thereby redirecting airflow to the bowel instead. In simulated colonoscopy, the insufflation button is optic based; and when activated, it starts to register already when the endoscopist’s finger is in close proximity of the button. Thus, the air insufflation button in simulated colonoscopy is much more sensitive than in real-life colonoscopy, resulting in endoscopists activating the button without meaning to.

The remaining seven metrics could be included in an aggregate score in one or both of the following two ways: (a) The metric was defined as essential for successful performance of the procedure and/or (b) the metric showed discriminatory ability between groups in this study.

- Essential metrics

Adopting a patient-safety approach [9], the abovementioned expert panel agreed upon which simulator-generated metrics were essential for performance of the procedure (content validity) and were required to pass the assessment by the use of Lawshe’s method [11, 12]. Three metrics reached a content validity ratio (CVR) of at least .99 and were categorized as essential for performance of the procedure. These were perforation and hazardous tension to the bowel wall (critical safety measures) and cecal intubation (critical procedure completion measure). The content validity index (CVI) was 1.0.

The items were defined as key actions necessary for successful performance of the procedure in the following way: the occurrence of perforation of the bowel wall resulted in resetting of the score to zero for the performed procedure (and thereby failing the attempt).

Application of hazardous tension to the bowel wall was a count outcome and resulted in a warning tone by the simulator. One occurrence was allowed since the warning served to enhance participants’ vigilance, while two or more warning sounds caused a reset of the score to zero.

Intubation of the cecum is globally recognised as the most important measure of quality in colonoscopy [13]. A reset of the score to zero was performed when the cecum was not reached during the procedure.

b. Metrics with discriminatory ability

Metrics showing significant differences between groups were used to create an aggregate score. This also applied for essential items with discriminatory ability.

### Assessment

The assessments in this study were performed in the research laboratory at the Corporate HR MidtSim Simulation Centre (Aarhus, Central Denmark Region).

All novel endoscopists received an introduction concerning how to hold and handle the colonoscope. They were introduced to the simulator and given 15 min of supervised training. Intermediate and experienced endoscopists were introduced to the simulator and given 10 min to try out the setting. Assessment commenced immediately after the introduction, and the two cases were performed successively without further practicing. All participants were allowed 10 min on each of the two cases. After that, the cases were terminated. Introduction and supervision of the initial training and assessment were performed by one expert endoscopist (CJ).

### Outcomes

The primary outcome of this study was an aggregate score created as an average of metrics with discriminatory ability divided by the time to reach the cecum and corrected for essential items. Secondary outcomes were seven computer-generated metrics: percentage of mucosa visualised, percentage of time spent with clear view, time to reach the cecum (measured in seconds), intubation of the terminal ileum (yes or no), percentage of time spent on looping, hazardous tension to the bowel wall (number), and occurrence of perforation (yes or no).

### Statistical methods

To detect a difference in the abovementioned aggregate score between novice, intermediate experience and expert with 80% power at the 5% significance level, a sample size of eight participants in each group was sufficient. Mean score, standard deviation, and difference of the mean score were used to calculate the sample size of this study. These assumptions were based on data from a previous study [5].

The simulator obtained metrics automatically. Discriminatory ability was identified using variance analysis for normally distributed metric data, Kruskal-Wallis test for not normally distributed data and Fisher’s exact test for categorical variables. Extensive loop formation was over dispersed; therefore, negative binominal regression was used. Hazardous tension to the bowel wall was a count outcome and was compared with Poisson regression.

In the first step, based on the metrics showing discriminatory ability, the aggregate score was calculated. The aggregate scores of the three study groups were compared with one-way analysis of variance (ANOVA) followed by pairwise comparisons with Tukey’s adjustment.

The three groups of participants were compared to set pass/fail standards (contrasting-groups method [14]). The consequences of these standards were explored to determine if any experienced endoscopists failed or novice endoscopists passed the assessment.

In the second step, the pass/fail score was adjusted according to the identified essential checklist items, resulting in a reset of the achieved score in one of the following instances: (1) perforation occurred, (2) the cecum was not reached, and (3) hazardous tension to the bowel wall was applied more than once.

Data were managed using REDCap electronic data capture tools hosted at Aarhus University [15, 16]. Statistical analysis was performed using Stata/MP 17 for Windows (version 17; StataCorp LP, Texas, USA). Differences were considered statistically significant at *p* values <0.05.

### Validation process

In accordance with the Standards for Educational and Psychological Testing, five sources of evidence were examined: evidence for test content, evidence based on response processes, evidence based on internal structure, evidence based on relations to other variables, and finally evidence for validity and consequences of testing [17, 18]. The contents of the assessment were evaluated by the abovementioned expert panel. To enhance validity in the response process, all information given to the study participants was provided by one expert endoscopist (CJ), who also supervised and observed all assessments on the simulator. To gather evidence on the internal structure of the assessment, Cronbach’s alpha was calculated based on the aggregate scores of the two cases performed. Evidence on relations to other variables was gathered by comparing the three groups (novice, intermediate, and expert endoscopists) based on the abovementioned aggregate score. Finally, consequences of the assessment were examined by setting a pass/fail standard, applying essential checklist items (abovementioned step 2) and exploring its effectiveness.

## Results

### Participant characteristics

Between 20 December 2019 and 20 July 2021, 24 participants were included in the present study. Of these, nine were male and 15 were female, aged 23 to 62. Seven participants were medical students, nine were residents in surgery, six were consultants in surgery, and two were nurse endoscopists. Five participants (one novice and four intermediates) had had 6 h of training in simulated colonoscopy prior to this study. Seven out of nine expert endoscopists were trained supervisors, performing 250 to 600 colonoscopies each year. Demographics can be seen in Table 1.

**Table 1:** Participant demographics

	**Gender, *n* (%)**		**Age, years**		**Colonoscopy experience**^a^	
	**Male**	**Female**	**Median**	**Range**	**Median**	**Range**
Novices (*n*=8)	2 (25)	6 (75)	24	23–29	0	0
Intermediates (*n*=8)	3 (38)	5 (62)	33	28–46	155	100–200
Experts (*n*=8)	4 (50)	4 (50)	43	38–62	3000	800–4000

^a^Number of colonoscopies performed

### Metrics

Seven metrics were obtained by the simulator. No perforations occurred.

Of the remaining six metrics, four showed a significant difference between the groups. Novices visualized a lower percentage of mucosa than intermediates and experts, they spent less time with clear view, needed more time to advance the colonoscope to the cecum, and were less successful in intubating the ileocecal valve. Further analysis showed that metrics measured in the novice group differed significantly from the other two groups. In none of these four metrics, a significant difference could be shown between intermediates and experts.

Although novices spent more time with extensive loop formation and applied hazardous tension to the bowel wall more frequently than intermediates and experts, the differences did not reach statistical significance. For further results, see Table 2.

**Table 2:** Simulator assessment metrics

Metrics	Novices (*n*=8) median (range)	Intermediates (*n*=8) median (range)	Experts (*n*=8) median (range)	*p* value
Mucosa visualized (%)				
Case 4	78.5 (19–88)	89 (80–92)	80.5 (72–91)	0.003†
Case 7	83.5 (17–87)	89.5 (82–94)	82 (72–88)	
Time with clear view (%)				
Case 4	94 (80–99)	94 (93–97)	96.5 (94–99)	0.027*
Case 7	92.5 (89–98)	94.5 (89–98)	97 (93–99)	
Time to cecum (s)				
Case 4	319 (297–545)	227 (144–360)	178 (122–268)	0.002†
Case 7	321 (240–591)	207 (164–322)	204 (103–258)	
Intubation of terminal ileum (*n*)				
Case 4	0/8	6/8	5/8	0.014‡
Case 7	5/8	7/8	7/8	
Extensive loop formation (s)				
Case 4	0 (0–12)	0 (0–9)	0 (0–4)	0.183§
Case 7	3.5 (0–75)	1 (0–8)	0 (0–19)	
Hazardous tension (n)				
Case 4	0 (0–4)	1 (0–2)	0 (0–1)	0.329^¶^
Case 7	1 (0–4)	0.5 (0–3)	1 (0–2)	
Aggregate score (points per minute)				
	8.9 (4.2–17.8)	23.1 (13.0–37.2)	31.1 (13.9–51.2)	<0.000*

The seven metrics of the Simbionix GI Mentor provided automatically for each of the two cases. The three groups were compared with: ^*^variance analysis, ^†^Kruskal-Wallis test, ^‡^Fisher’s exact test, ^§^negative binominal regression, or ^¶^Poisson regression

### Aggregate score—step one

The aggregate score was calculated on the basis of the four discriminatory metrics using the following Eq. [5]:

$$\left(\left((\mathrm{mucosa\,visualized},\mathrm{ \%})+(\mathrm{clear\,view},\mathrm{ \% })+(\mathrm{ileum\,intubation}:\mathrm{yes}=100\mathrm{\%},\mathrm{ no}=0\mathrm{\%})\right)/3\right)/\mathrm{time\,to\,cecum\,in\,minutes }=\mathrm{ aggregate\,score}$$

The scores ranged from 4.2 to 51.2. The median (range) aggregate score was 8.9 (4.2–17.8) for novices, 23.1 (13.0–37.2) for intermediates, and 31.1 (13.9–51.2) for expert endoscopists (see Table 2). The difference in score between novices and the other two groups was significant (*p*=0.01). Although expert endoscopists had a higher score, the difference between intermediates and experts did not reach statistical significance (*p*=0.40).

The internal consistency reliability of the two measurements of the aggregate score was calculated and showed a Cronbach’s alpha=0.86.

**Fig. 2:**
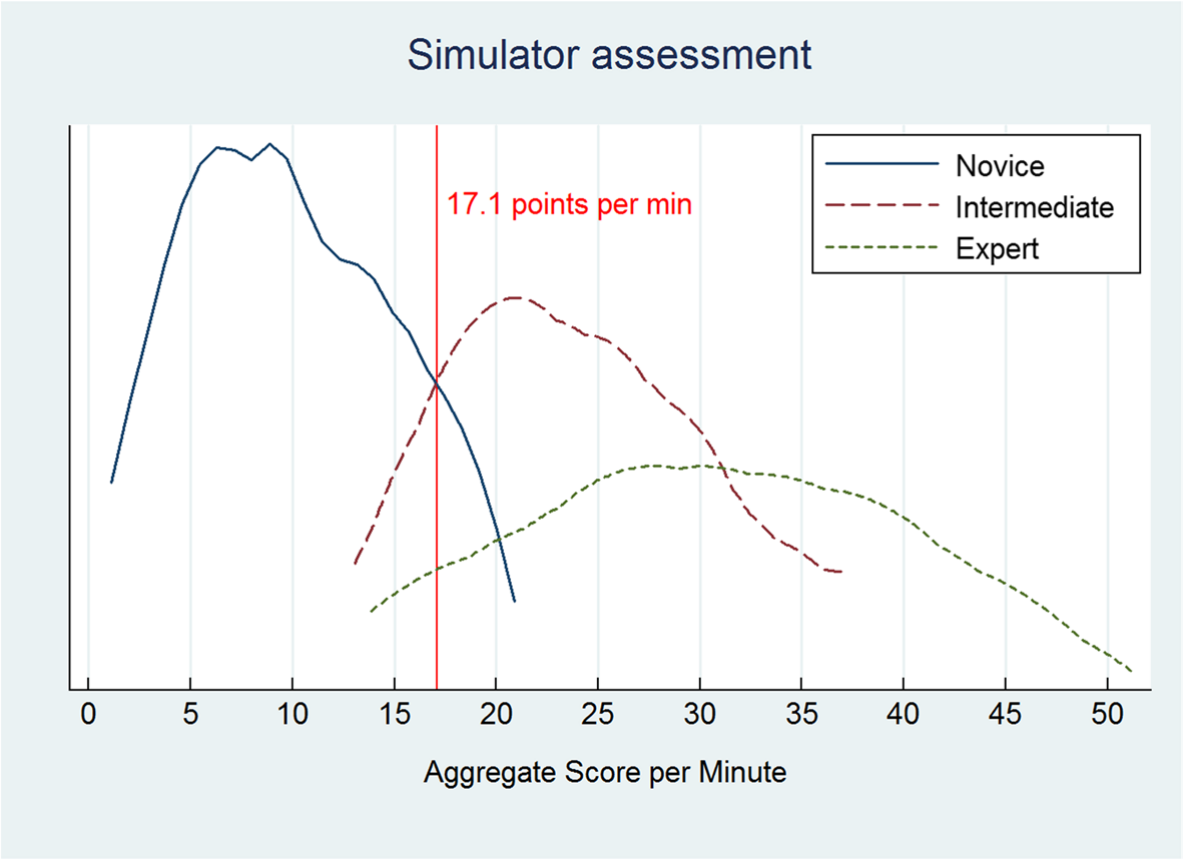
Aggregate score per minute for each of the three study groups (novice, intermediate, and expert endoscopists). Results show the pass/fail standard at the intersection of the novice and intermediate distributions at 17.1 points per minute

Figure 2 illustrates aggregate scores for the three study groups. Based on the results, the pass/fail score was defined as the intersection between the distributions for novices and intermediates, viz. 17.1 points. One expert and one intermediate failed, while one novice passed the assessment. No pass/fail standard could be set at the intersection between intermediate and experts because the aggregate scores in these two groups did not differ significantly.

### Essential checklist items—step two

#### Perforation

No perforations occurred during any of the simulated colonoscopies in this study.

#### Cecal intubation

Four participants (all of them novices) did not reach the cecum in one or both performed simulated cases. These four participants did not reach the pass/fail score of 17.1 points per minute in the first step.

#### Hazardous tension

Nine participants (four novices, three intermediates, and two experts) exceeded the permissible amount of hazardous tension to the bowel wall in one or both performed cases, and failed the assessment. Of these, four novices and one intermediate did not reach the pass/fail score of 17.1, while two intermediates and two experts failed the assessment solely because of the applied tension to the bowel wall.

When aggregate score, perforation, hazardous tension, and cecal intubation were all taken into account, one novice, five intermediates, and five experts passed the assessment.

## Discussion

In the present study, we found four metrics with the ability to discriminate between novices and more competent endoscopists, but not between intermediate and expert endoscopists.

These four metrics (percentage of visualized mucosa, time to reach the cecum, percentage of time spent with clear view, and ability to intubate the terminal ileum) are key performing indicators for good endoscopy practice, which is underpinned by the systematic review of 13 studies by Ansell et al. who found the same metrics to be of importance in assessing simulated colonoscopy skills across several different simulators [19]. However, they did not provide a pass/fail standard.

In our study, the Simbionix colonoscopy simulator was able to differentiate novices in colonoscopy from more experienced endoscopists. However, the simulator could not distinguish between different levels of competency once a certain expertise in colonoscopy was achieved. This was also seen in a recent study from Oberoi et al. [20].

A plausible reason for this inability might be the simulator’s lack of realism [21]. The simulator registers a certain set of basic movements and actions, for example, tip steering, insufflation, and insertion depth. Other more refined real-life actions like torque steering, insertion force, changes in patient positioning, or application of pressure on the abdomen are either not registered, not possible, or do not alter the simulation outcome. Thus, if not registered by the simulator, the more refined actions characterising endoscopic expertise will not be recognized. Consequently, if not included in the simulator’s repertoire, these actions cannot be taught and, thus, more competent endoscopists experience reduced benefit of and performance in simulation training. Several studies point in that direction [4, 22, 23]. In one study, the learning effect from the simulator ceased after 80 real-life colonoscopies [24].

In the present study, we tried to find a pass/fail standard that marks the transition from “able to perform on the simulator without the potential of further simulation-based learning” to “competent for work-based learning”. Basically, we tried to compare two very different settings: simulated colonoscopy and real-life colonoscopy. In order to spare patients for unduly strain, we measured performance of novices against experienced endoscopists in a simulated setting. There is no evidence supporting the assumption that a pass/fail standard on the simulator is equivalent to being able to progress to work-based learning. The same concern applies to the question whether assessment of critical safety items translates to increased patient safety. Further research is needed to address these questions.

We defined an aggregate score of 17.1 points per minute and passing a set of essential checklist items as the pass/fail standard. However, a single passed assessment does not necessarily equal consistent performance at the required competency level. A recent study in orthopaedic surgery indicates that each trainee will reach a plateau of consistent performance when training simulated hip surgery [25]. They propose the use of a plateau score rather than spent training time or number of repetitions as criteria for competency. This approach may be advantageous in colonoscopy training as well. However, the present study did not set out to investigate the plateau score. Future studies should be conducted to show in what way a pass/fail score could be applied, and whether our proposed score can be utilized to mark a plateau of consistent performance [25].

Another limitation is the cutoff threshold for two of the three essential checklist items. For obvious reasons, a simulated procedure ending in a perforation needs to be assessed as failure. However, evaluation of the other items is more complicated. The critical standard for hazardous tension (one instance is acceptable, more than one results in failure) is an arbitrarily set standard. As there is no measure of hazardous tension to the bowel wall in real-life colonoscopy, the cutoff threshold was reached by discussion in the abovementioned expert panel. Regarding cecal intubation, endoscopists are recommended to reach the cecum in ≥90% of the cases [13]. We decided to set the standard for cecal intubation to 100% (except cases with tumour stenosis) for two reasons: (1) by setting the standard to 100%, assessment was possible for each procedure and (2) requiring high standards for performance in a simulated setting might prevent adverse events in an actual patient situation [9].

Also, a significant limitation is the preclusion of certain metrics, and the simulator provides. For example, the metric “discomfort,” which is an important measure of proficiency: in clinical practice, increased endoscopist expertise results in lower pain levels [26–28]. However, as discomfort measurement in simulated procedures is different than in real-life colonoscopy, the metric “discomfort” cannot be used in its actual form. The same problem applies to simulated polypectomy. Polypectomy is a highly technical skill and is associated with a higher risk for hemorrhage and perforation [29]. Polypectomy skills would be very important to include in a competency assessment, yet the very basic simulation of polypectomy on the Simbionix does not mirror the real skill and can therefore not be used for assessment in its current state.

An important limitation of this study is the inclusion of participants based on the number of colonoscopies they had performed. Competency in colonoscopy is not only a matter of the quantity of procedures performed; more important is a group of key performance indicators like adenoma detection rate, cecal intubation rate, and withdrawal time [13]. Furthermore, other factors may also need to be taken into consideration such as communication and teamwork, situation awareness, leadership, and decision making; the so-called endoscopic non-technical skills (ENTS) [30, 31]. The participants in this study may have been grouped otherwise if key performance indicators or non-technical skills had been taken into account as well.

The scope of the present study was to establish competency levels in simulation-based training; however, it is well known in the transfer literature that simulation-based training should be integrated in postgraduate specialist training by including the three transfer inputs; trainee characteristics, training design, and work environment [32]. The present study set out to investigate the training design based on trainee characteristics. Subsequently, the work environment should be taken into account. Here, ecological validity and the context are emphasized in the medical education literature [33]. In the present study, our simulation-based findings did not directly address the ecological validity and implementation in the context of a real-life setting of an endoscopy training programme in workplace-based training. Future studies could investigate alignment between the proposed simulation-based training and workplace-based colonoscopy training, thereby enabling transfer [34].

## Conclusion

The metrics in the GI Mentor simulator are able to discriminate between novices and more experienced endoscopists but discerned no statistically significant difference between intermediate and expert endoscopists. The colonoscopy simulator is an important and feasible training tool that can be used for assessment in an endoscopy training program at the lower end of experience. We propose an aggregate score of 17.1 points as pass/fail score for assessment. Also, standards of three essential checklist items (perforation, cecal intubation, and hazardous tension) should be met for passing the assessment. We recommend future studies to confirm our findings.

## Data Availability

The datasets used and analyzed during the present study are available from the corresponding author upon reasonable request.

## Abbreviations

ANOVA: Analysis of variance
CVI: Content validity index
CVR: Content validity ratio
ENTS: Endoscopic non-technical skills
GI: Gastrointestinal
SD: Standard deviation
