# Correction: Development and validation of a simulation-based assessment tool in colonoscopy

**DOI:** 10.1186/s41077-023-00272-1

**Published:** 2023-12-20

**Authors:** Claudia Jaensch, Rune D. Jensen, Charlotte Paltved, Anders H. Madsen

**Affiliations:** 1 Surgical Research Department, Regional Hospital Gødstrup, Herning, Denmark; 2 Department of Clinical Medicine, Aarhus University, Aarhus, Denmark; 3 Corporate HR MidtSim, Central Region of Denmark, Aarhus, Denmark; 4 Surgical Department, Regional Hospital Gødstrup, Herning, Denmark

**Correction: Advances in Simulation 8, 19 (2023)**

**https://doi.org/10.1186/s41077-023-00260-5**

Following publication of the original article [1], the authors request to update the Competing interests section as follows:

“The authors declare that Rune D. Jensen is an Associate Editor of the journal."
